# Integrating HIV and hypertension management in low-resource settings: Lessons from Malawi

**DOI:** 10.1371/journal.pmed.1002523

**Published:** 2018-03-07

**Authors:** Pragna Patel, Colin Speight, Alice Maida, Fleetwood Loustalot, Denise Giles, Sam Phiri, Sundeep Gupta, Pratima Raghunathan

**Affiliations:** 1 Center for Global Health, Centers for Disease Control and Prevention, Atlanta, Georgia, United States of America; 2 The Lighthouse Trust, Kamuzu Central Hospital, Lilongwe, Malawi; 3 National Center for Chronic Disease Prevention and Health Promotion, Centers for Disease Control and Prevention, Atlanta, Georgia, United States of America; 4 University of North Carolina School of Medicine, Department of Medicine, Chapel Hill, North Carolina, United States of America; 5 University of Malawi, College of Medicine, School of Public Health and Family Medicine, Department of Public Health, Lilongwe, Malawi

## Abstract

Pragna Patel and colleagues describe the implementation of a hypertension management model for HIV-infected people in Malawi.

**Summary points**

- Hypertension is the leading risk factor for cardiovascular disease, the most common cause of death worldwide.
- In 2011, the United Nations declared that lessons learned from national human immunodeficiency virus (HIV)/acquired immunodeficiency syndrome (AIDS), tuberculosis, and malaria programs in low- and middle-income countries should be harnessed for effective integration of communicable and noncommunicable disease initiatives.
- HIV clinical service delivery facilities offer a platform on which to enhance care by offering noncommunicable disease interventions.
- Given Malawi’s high prevalence of hypertension, with an estimated one-third of adults in the country affected, we sought to incorporate hypertension management in HIV care delivery sites using standardized treatment protocols and patient registries to monitor treatment outcomes.
- The overall aim was to create a reproducible and scalable hypertension management model for HIV-infected persons that is cost effective and that can be used to inform national scale-up of hypertension and other chronic disease care models.
- We describe pertinent issues that were raised during the design and implementation of the program, particularly important for the enhanced care of HIV-infected persons.

## Introduction

Hypertension is the leading risk factor for cardiovascular disease (CVD), the most common cause of death worldwide [1]. Although sub-Saharan Africa (SSA) is among the last regions in the world to undergo the epidemiologic transition of mortality from infectious disease to noncommunicable disease (NCD), there is a high dual burden of communicable and noncommunicable diseases in this region [2,3]. Currently, acquired immunodeficiency syndrome (AIDS) is the leading cause of death in SSA [2]. However, with the successful expansion of access to antiretroviral therapy (ART) in SSA, dramatic reductions in AIDS-related morbidity and mortality are expected, as seen in high-income countries [4]. As persons living with human immunodeficiency virus (PLHIVs) in SSA gain access to ART and achieve virologic suppression, they will live longer and experience a similar life expectancy to HIV-uninfected persons [4]. Thus, comorbidities, such as CVD, will become increasingly prevalent [5].

PLHIVs are at an increased risk for CVD because of an increased prevalence of traditional risk factors and nontraditional risk factors (e.g., inflammation) as well as effects of specific antiretroviral drugs [6]. Given that PLHIVs are at higher risk for CVD [7], the treatment and control of a high-burden, treatable risk factor such as hypertension are important as an entry point to a more comprehensive approach to CVD management. Hypertension is common in SSA, with prevalence estimates in the general adult population ranging from about 15% to 40% [3]. However, the majority of persons with hypertension (60%–90%) are unaware of their condition, and very few (<5%) have achieved blood pressure control [8]. Therefore, there is a demonstrated need for hypertension screening and control programs in the region.

## The setting: Malawi

Malawi has a government-funded national healthcare service that is free to all Malawians. Delivery of healthcare occurs at 3 levels: health centers at the local level, regional/rural hospitals one level up, and district hospitals at the highest level. Because of low resources, investigations are limited, and diagnosis is largely based on clinical presentation. Most laboratory, imaging, and testing facilities are often only available at the major district hospitals. Malawi has very few doctors; therefore, clinical officers (who are trained for a minimum of 4 years and are very experienced practitioners) and medical assistants (who are trained for a minimum of 3 years) are the main healthcare cadre [9].

Malawi has one of the highest HIV prevalence rates (10.6%) among persons aged 15–64 years in the world, and 33% of Malawians aged 25–64 years have high blood pressure (BP) [3]. The President’s Emergency Plan for AIDS Relief (PEPFAR) was responsible for the rapid growth of ART clinics in Malawi with decentralization of services, which have provided life-saving ART to over 600,000 PLHIVs at 650 sites nationwide. Although quality HIV programs exist, there remains a large unmet need in Malawi to improve the quality of healthcare services, promote early detection, and optimize clinical management of hypertension to reduce illness and avoid premature deaths. Additionally, while costs are low for routine BP screening during clinical visits, the HIV clinics in Malawi were not routinely offering these services. The extensive PEPFAR-supported clinic network in Malawi illustrated the potential programmatic reach for the management of other chronic diseases using HIV as a platform for service delivery. For these reasons, the Centers for Disease Control and Prevention (CDC) partnered with Malawi’s Ministry of Health (MOH) and the Lighthouse Trust, the largest provider of ART in Malawi, to integrate hypertension screening and treatment into existing PEPFAR-funded HIV care clinics. The Lighthouse Trust provides services to 25,000 PLHIVs of all ages and plays a pivotal role in piloting service delivery models, introducing curricula on behalf of the national HIV program to build capacity. It has its own procurement mechanism, which facilitated and ensured the availability of antihypertensive medications.

## Ethical oversight

The investigation followed the guidelines of the United States Department of Health and Human Services regarding protection of human participants. The study protocol was reviewed according to the CDC, the Malawi MOH, and the Lighthouse Trust human participants review process and was determined to be nonresearch.

## Health systems strengthening approach

The integrated HIV and hypertension screening and management program was implemented using a health systems strengthening approach and leveraging the public health approach, which was successfully employed for the global HIV response [10]. According to WHO, health systems consist of 6 building blocks that reflect the basic functions they should carry out regardless of how they are organized: service delivery; health workforce; information; medical products, vaccines, and technologies; financing; and leadership and governance (stewardship) [11]. This framework was developed to address health inequalities worldwide.

The key elements of a successful hypertension management program reach beyond clinical care delivery and require agreement and engagement from public health leaders, governments, and healthcare providers, as well as broad policies to support integration with HIV care. The main areas of our focus were policy, systems strengthening, innovation, education, and monitoring/evaluation (Table 1). Ensuring the consistent availability of low-cost, high-quality medications by improving existing supply chains or developing new procurement methods is paramount [12]. As consistent access to medications was available at our sites, we focused attention to optimal hypertension care delivery (Table 1) for low-resource settings [13].

**Table 1:** Recommended elements for successful hypertension and human immunodeficiency virus (HIV) management integration.

Category	Recommended elements	Considerations for the HIV-infected population	Used in Malawi hypertension-HIV program
Policy	Population-level interventions (sodium reduction) National clinical guidelines	Guidelines that consider drug interactions for hypertension treatment	Developed algorithms for hypertension treatment that would minimize interactions with antiretrovirals
System strengthening	*Service delivery*: Standardized assessment Simplified management protocols Blood pressure measurement devices CVD risk estimation Task shifting Training Clinical mentorship *Supply chain*: Improved medication procurement and sustained availability Access to fixed-dose combination medications *Community engagement*: Linkage to care Community health workers Leveraging existing infrastructure/systems	Benefit of angiotensin-converting enzyme inhibitors Leverage HIV medication supply chain improvements Enhanced adherence to complicated regimens to treat HIV and co-morbidities Enhance PEPFAR service delivery	*Service delivery*: Standardized assessment Simplified management protocols Blood pressure measurement devices Task shifting Training Clinical mentorship *Supply chain*: Improved medication procurement and sustained availability Leveraging existing infrastructure/systems
Innovation	Promotion and use of available technology Electronic health records mHealth Telemedicine Point-of-care laboratory testing Improve availability of fixed-dosed combination medications (e.g., medications with 2 active ingredients)		Electronic health records
Education	*Community*: Mass media campaigns Population-level health promotion *Clinical*: Training of healthcare workers during formal education and in clinical practice Culturally tailored educational materials	Leverage platforms for HIV education to include chronic disease education	*Clinical*: Training of healthcare workers during formal education and in clinical practice Culturally tailored educational materials
Monitoring/evaluation	Tracking/indicators Registries/cohort monitoring Clinical feedback loop (chart reviews, line listing, clinic-level control estimates, etc.) Performance metrics for clinicians Program evaluation using process and outcome indicators	Track HIV-specific indicators (e.g., ART adherence)	Tracking/indicators Registries/cohort monitoring Clinical feedback loop (chart reviews, line listing, clinic-level control estimates, etc.) Performance metrics for clinicians Program evaluation using process and outcome indicators
Optimal hypertension care delivery	Standardized screening and diagnosis using certified and calibrated devices Education about disease prevention and counseling using culturally appropriate lifestyle modifications Standardized treatment protocols Task shifting Cohort monitoring		Standardized screening and diagnosis using certified and calibrated devices Education about disease prevention and counseling using culturally appropriate lifestyle modifications Standardized treatment protocols Task shifting Cohort monitoring

Abbreviations: ART, antiretroviral therapy; CVD, cardiovascular disease; mHealth, mobile health; PEPFAR, President’s Emergency Plan for AIDS Relief. Cohort monitoring: use of longitudinal patient registers or electronic medical records systems to regularly monitor progress and outcomes.

## The Lighthouse HIV-hypertension integrated model

We initiated a pilot program at 2 high-volume HIV clinics operated by the Lighthouse Trust in Lilongwe, Malawi, as a demonstration project to inform modifications to our integrated hypertension and HIV model and to identify challenges that warrant consideration. The aims of the project were to (1) integrate hypertension screening and treatment in HIV care clinics, (2) optimize hypertension treatment outcomes using standardized hypertension treatment protocols and cohort monitoring, and (3) create a reproducible and scalable hypertension management model that is cost-effective and can be used to inform national scale-up and other chronic disease care models.

We encountered significant design and policy issues, which included the development of standardized hypertension treatment protocols specific to the HIV population and consistent with the Malawi Standard Treatment Guidelines, establishment of an adequate referral network for complicated and resistant cases, and consensus on the need for routine laboratory screening and cardiovascular disease risk assessment in the context of limited resources. Below, we discuss each of these issues in more detail and explain the rationale for our decision-making process.

### Developing treatment guidelines

**Fig 1:**
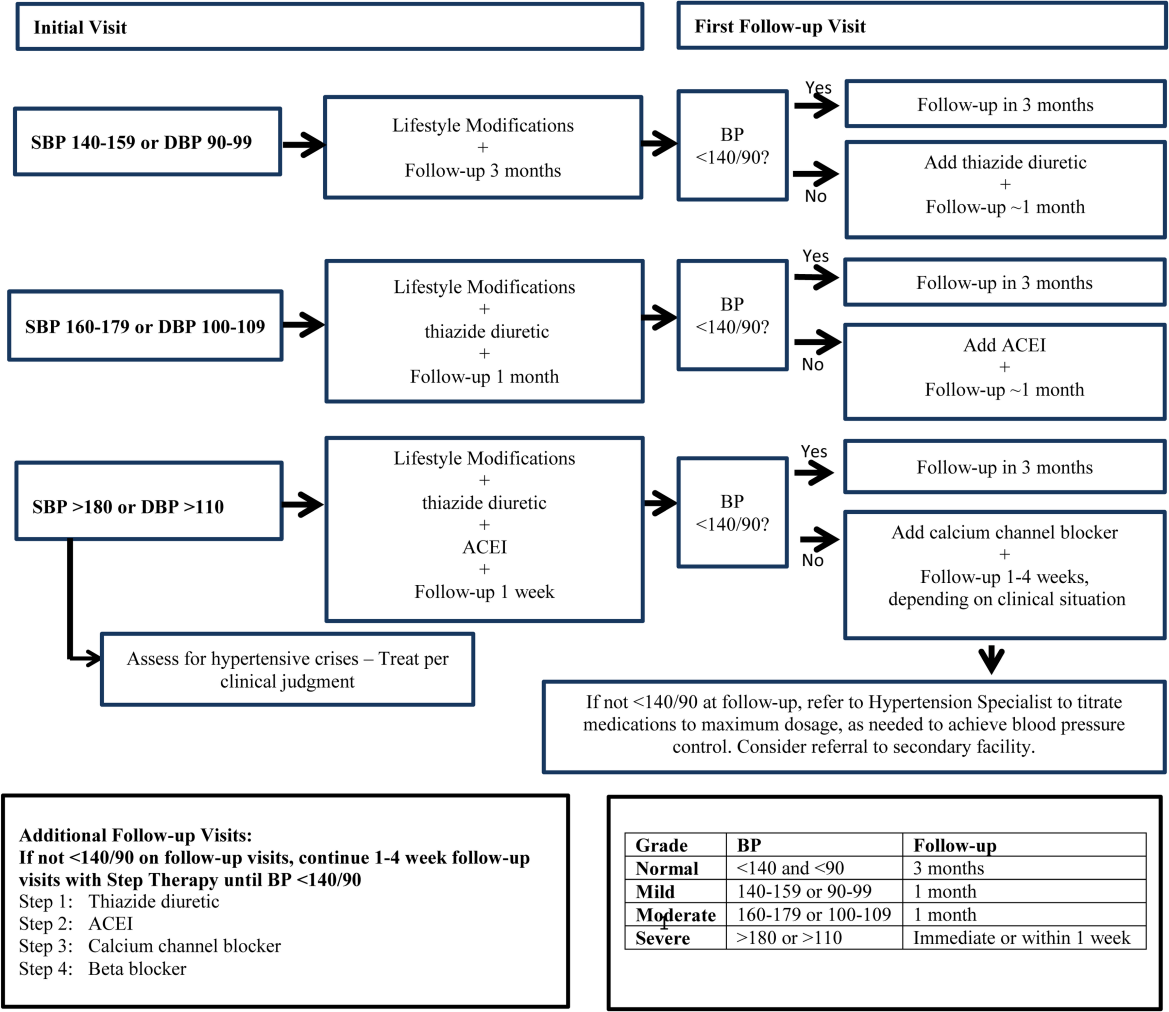
Suggested algorithm for treatment of hypertension among human immunodeficiency virus (HIV)-infected persons. ACEI, angiotensin-converting enzyme inhibitor; BP, blood pressure; DBP, diastolic blood pressure; SBP, systolic blood pressure.

In developing standardized treatment protocols, drug-drug interactions between antihypertensive medications and ART warrant careful consideration. We aimed to minimize the number of patients likely to encounter significant interaction with non-nucleoside reverse transcriptase inhibitor (NNRTI)-based or protease inhibitor (PI)-based regimens, as in SSA virtually all regimens in use include one or the other. In Malawi, the standard treatment guidelines recommend thiazide diuretics as first-line treatment for hypertension and calcium channel blockers as second-line, with no special provisions for PLHIVs. Calcium channel blockers may interact with NNRTIs and PIs, requiring dose adjustment [14], and angiotensin-converting enzyme inhibitors (ACEIs) have been associated with anti-inflammatory effects [15]. Given the potential drug interaction with calcium channel blockers and potential benefit of ACEIs, we used ACEIs as second-line agents for the treatment of hypertension. To prevent electrolyte imbalances, we suggested low-dose therapy of each class of medication with addition of another class rather than titration to maximum dose of current medication for patients with uncontrolled BP. To support the expansion of this model to austere clinical sites, we have developed a recommended algorithm for hypertension treatment for patients who are stable on ART (Fig 1). Furthermore, we received permission for the modification of the hypertension national guidelines from the Malawi Ministry of Health to accommodate necessary changes for the treatment of hypertension among PLHIVs.

### Referral network for those with complicated hypertension

Although the majority of hypertension patients will have essential hypertension, the possibility of secondary and resistant hypertension should be assessed in patients with poor response to antihypertensive therapy despite good adherence, in previously stable patients with worsening hypertension, and in patients with stage 3 hypertension (BP > 180/110) and significant target organ disease [16]. The creation of a referral network, which is a type of integrated health service, can facilitate directing persons to appropriate centers as indicated and is thus recommended, as PLHIVs may have additional factors that warrant consideration by a specialist. The use of technology, such as telemedicine, to access clinical specialists for those requiring advanced assessment may also be considered for those communities or clinic populations with inadequate access to care or in geographically isolated areas. We decided to refer patients to an academic physician, who is a local expert in chronic diseases and thus a hypertension specialist, after evaluation by the clinic medical director, who is a certified physician.

### Laboratory monitoring

Renal insufficiency, hypokalemia, and other complications may occur with the addition of antihypertensive medications to ART and can be detected with laboratory screening. Laboratory monitoring for those on antihypertensive therapy may not routinely be performed in SSA; however, it may be available for certain clinical indications, such as for patients who develop signs and symptoms that suggest an electrolyte abnormality. If the clinical site is able to conduct phlebotomy and separate serum for laboratory investigations and funding is available to pay for laboratory costs, chemistry panels, which include creatinine and potassium measurements, should be conducted. Testing for creatinine and potassium is recommended at baseline and at the 1-month follow-up visit, particularly for patients starting thiazide diuretics and ACEIs. The need for routine laboratory monitoring is unclear and in extremely resource-poor settings may be impractical to implement. Lack of access to laboratory monitoring should not preclude screening and treatment of hypertension, as the number of patients who experience electrolyte abnormalities is small. Syndromic surveillance can be instituted to assess for potential abnormalities [17]. In our project, we opted to forgo laboratory screening given the challenges with implementation, lack of resources, and expected relatively low yield.

### Cardiovascular disease risk assessment

HIV-specific factors such as low CD4^+^ cell counts and exposure to specific antiretrovirals (ARVs) have been found to be independent risk factors for incident CVD and can increase CVD risk as much as 50%–80% among PLHIVs [7]. Most CVD risk assessment tools tend to underestimate CVD risk among PLHIVs [18]. Therefore, use of CVD risk assessment models may fail to identify substantial numbers of PLHIVs with elevated CVD risk who could potentially benefit from additional medical treatment. Nonetheless, in a low-resource setting, CVD risk assessment can be used to prioritize treatment for those who would benefit most among those PLHIVs who have achieved virologic suppression. We did not institute CVD risk assessment, as further studies on CVD risk assessment among PLHIVs are warranted.

## Implementation issues

We encountered key implementation issues, which included training of a cadre of healthcare providers to screen for and treat hypertension among PLHIVs; integrating care for an additional disease entity without overwhelming the present infrastructure in terms of patient load per provider, clinic wait times for patients, and quality of care; and developing routine monitoring and evaluation systems. The medical director provided regular monthly classroom trainings to approximately 25 nurses and clinical officers who staffed the 2 clinics. These trainings were updated periodically to address issues that were of concern; for example, early in implementation, we noted that patients diagnosed with hypertension were not filling their prescriptions right away. These types of issues were addressed at weekly meetings with the medical director, and solutions were incorporated into subsequent trainings. To avoid disrupting clinic flow and ensure a manageable workload, we used a phased screening approach by gradually decreasing the age threshold of eligible persons. Process and outcome indicators developed for the project are summarized in Table 2. Registries can be used to generate performance metrics for healthcare providers as well as inform clinical feedback to identify patients who need follow-up care. Therefore, we developed a hypertension-specific module for the Malawi electronic medical record system that provided screening and management prompts for clinical decision support and facilitated data collection, including process indicators, and analysis for ongoing program evaluation and quality of care improvement. We were particularly concerned about the additional burden of HIV medications affecting adherence to ARVs; therefore, we collected data on adherence, which was already being measured as part of the HIV program.

**Table 2:** Example core process and outcome indicators for an integrated hypertension/human immunodeficiency virus (HIV) clinical management program examined at 6-month intervals.

Domain	Type	Description	Numerator	Denominator
**Hypertension control**	**Outcome**	Percentage of HIV patients 18 to 85 years of age with a diagnosis of HTN and whose blood pressure was adequately controlled (<140/90) during the measurement period	Patients from the denominator with last blood pressure measurement with systolic blood pressure less than 140 mm Hg and the diastolic blood pressure less than 90 mm Hg	All patients 18 to 85 years of age with a diagnosis of HTN during the measurement period who received treatment for at least 3 months
**Clinical quality**	**Process**	Percentage of HIV hypertensive patients aged 18 to 85 years with a blood pressure reading of greater than 140/90 mm Hg who were treated as outlined in the standardized treatment protocol	Number of patients aged 18 through 85 years and with hypertension and blood pressure greater than 140/90 mm Hg who received lifestyle modification and/or treatment	Number of hypertensive patients aged 18 through 85 years and with blood pressure greater than 140/90 mm Hg
**Clinical quality**	**Process**	Percentage of electronic health record alerts with a documented repeat blood pressure at same visit if the first reading is above 140/90 mm Hg	Number of documented repeat blood pressure readings at the same visit if the first reading is above 140/90 mm Hg	Number of electronic health record alerts indicating first reading is above 140/90 mm Hg
**Clinical quality**	**Process**	Percentage of treated hypertensive patients with acceptable ART adherence (>95%)	Number of hypertensive patients with ART adherence above a specified threshold	Number of HIV patients with HTN on antihypertensive medications
**Clinical quality**	**Outcome**	Percentage of HIV/HTN patients lost to follow-up from care and treatment	Number of HIV/HTN patients lost to follow-up from care and treatment	Number of HIV/HTN patients alive and on treatment
**Healthcare delivery**	Cost*	Resources used by HIV patients with hypertension during the measurement period	Total standard cost and service frequency counts for all services, which accounts for service usage, for which the organization has paid or expects to pay for the eligible population during the treatment period, reported by age, gender, and risk group	HIV patients 18 through 85 years of age with a diagnosis of hypertension

Abbreviations: ART, antiretroviral therapy; HTN, hypertension.

*Detailed cost parameters are not provided as they may vary across programs

## Potential to improve HIV outcomes and access hard-to-reach populations

Integrated service delivery and decentralization of care into primary health facilities and communities can improve outcomes among PLHIVs. Expansion in ART coverage, improvement in retention, and reduction in mortality have all been demonstrated in programs that have successfully integrated HIV services into clinics for maternal, newborn, and child care as well as tuberculosis treatment and opiate substitution therapy [19].

The integration of HIV and NCD management may particularly improve access to care for adult males in SSA, a population that has remained elusive despite PEPFAR’s efforts to expand ART [20]. Also, the integration of HIV and NCD services may reduce stigma associated with HIV, especially if these clinics eventually serve the general uninfected population [20]. A key to improving access to ART and HIV outcomes may be to use the platforms that have been developed for HIV care to build health systems that are available to all, thereby addressing the double burden of HIV and NCDs effectively both at an individual as well as a population level. Further research and evaluation of integrated care models are needed to inform efforts to use limited healthcare resources effectively [21].

## Discussion

The hypertension management model described here is consistent with the WHO’s framework for health systems strengthening and considers the 6 building blocks that make up the health system [11]. In addition, we promote the use of evidence-based tools and practices to improve BP control.

In our demonstration project, at 12 months, of 29,359 individuals screened, 11% were newly diagnosed with hypertension, and 85% of those with hypertension received treatment per standardized protocols. Among persons with mild and moderate hypertension, BP control rates were 38% and 30% after 6 months of treatment, respectively (Table 3). The additional drug burden for hypertension control did not affect adherence to HIV medications. We did not see any electrolyte abnormalities among persons prescribed antihypertensive medications during the course of the project. This project successfully integrated hypertension screening and management into routine HIV service delivery at 2 Lighthouse Trust HIV clinics; however, expansion within Malawi to government-funded sites is challenging because of the inconsistent supply of medications for these types of facilities. Program evaluation is currently underway.

**Table 3:** Postimplementation program evaluation data for 2 Lighthouse Trust pilot sites in Lilongwe, Malawi, February 2015 to June 2016.

Characteristic	Total
*Screening parameters*	
Number of persons screened*†	29,359
Median age (IQR)	38 (32–45)
Male gender (%)	11,794 (39)
*Diagnosis parameters*	
Number newly diagnosed with hypertension (%)‡	3,448 (11)
Mild (SBP 140–159 or DBP 90–99)	1,619 (47)
Moderate (SBP 160–179 or DBP 100–109)	514 (15)
Severe (SBP ≥ 180 or DBP ≥ 110)	1,315 (38)
*Treatment parameters*	
Number of hypertensive patients started on treatment or given lifestyle advice (%)	2,915 (85)
Number of all hypertensive patients on pharmacologic treatment for hypertension (%)ǁ	1,681 (49)
*Control parameters*	
Of those on treatment for 3 months, number with normal blood pressure at last visit (%)	240 (22)
Of those on treatment for 6 months, number with normal blood pressure at last visit (%)	229 (26)
*Adherence parameters*	
Number of hypertensive patients on treatment with at least 95% antiretroviral adherence	1,165 (80)
Number of patient without hypertension with at least 95% antiretroviral adherence	20,073 (79)

Abbreviations: DBP, diastolic blood pressure; SBP, systolic blood pressure.

*A phased approach was used at the Martin Preuss Clinic; therefore, the program was considered fully operational in June 2015, and the evaluation period extended to 12 months from this date to June 2016.

†All patients were screened with an automated sphygmomanometer.

‡The definition of a new diagnosis of hypertension (HTN) was based on Malawi Standard Treatment Guidelines (fourth edition): SBP ≥ 140 mm Hg or DBP ≥ 90 mm Hg measured on 2 consecutive visits or severe HTN (SBP ≥ 180 mm Hg or DBP ≥ 110 mm Hg) on a single visit.

ǁProvision for treatment: all persons with moderate or severe HTN were eligible for treatment. For those with mild HTN, only lifestyle modifications were suggested unless the individual had one cardiovascular disease (CVD) risk factor (current smoker, diabetes mellitus [DM], history of CVD, or history of CVD in a first-degree relative).

Site-specific program costs associated with this add-on intervention will provide an estimate of the average treatment cost per patient, and program cost-effectiveness analysis will help assess whether the health return attributable to the intervention may be justified. Such cost-effectiveness information may be useful in advocating for replication of the model among civil society stakeholders and global policy makers, potentially leading to expanded public health impact through the SSA region. Consistent access to medications for hypertension was available at our sites, which may not be the case in many clinics, so this model is generalizable to clinics with similar infrastructure in place. Hypertension and HIV integrated care will need continued investments in supply-chain management because stock-outs of antihypertensive medications commonly occur for the publicly funded clinics in Malawi. In Malawi, these drugs are offered free of charge; however, this creates challenges for public health officials who need to distribute limited resources to a number of programs, especially as support for HIV medications starts to shift from PEPFAR to national ministries. Innovative approaches to paying for medications should be sought. For example, regional procurement mechanisms may reduce overall costs, with bulk procurement rendering medications more available and affordable [12]. In addition, we were not able to implement care for comorbidities such as diabetes given limited resources and staff. Finally, training a cadre of healthcare workers to provide both chronic multidisease and HIV care may pose distinct challenges given limited numbers of available personnel.

We hope that this program will help to inform the larger effort to improve health systems and integrate HIV and NCD services for improved care. As outlined in the NCD global action plan, this project directly relates to the priority action of member states to examine opportunities for HIV-NCD care integration not only to manage NCDs more effectively but also to enhance the care of PLHIVs as they age and live longer due to improve ART coverage throughout the world [22].

## Abbreviations

ACEI: angiotensin-converting-enzyme inhibitor
AIDS: acquired immunodeficiency syndrome
ART: antiretroviral therapy
ARV: antiretroviral
BP: blood pressure
CDC: Centers for Disease Control and Prevention
CVD: cardiovascular disease
HIV: human immunodeficiency virus
HTN: hypertension
MOH: Ministry of Health
NCD: noncommunicable disease
NNRTI: non-nucleoside reverse transcriptase inhibitor
PEPFAR: President’s Emergency Plan for AIDS Relief
PI: protease inhibitor
PLHIV: person living with human immunodeficiency virus
SSA: sub-Saharan Africa
